# Is a timely assessment of the hematocrit necessary for cardiovascular magnetic resonance–derived extracellular volume measurements?

**DOI:** 10.1186/s12968-020-00689-x

**Published:** 2020-11-30

**Authors:** Mao-Yuan Su, Yu-Sen Huang, Emi Niisato, Kelvin Chow, Jyh-Ming Jimmy Juang, Cho-Kai Wu, Hsi-Yu Yu, Lian-Yu Lin, Shun-Chung Yang, Yeun-Chung Chang

**Affiliations:** 1 Department of Medical Imaging, National Taiwan University Hospital, No.7, Chung-Shan South Road, Taipei, 100 Taiwan; 2 Siemens Healthcare Limited, Taipei, Taiwan; 3 Siemens Medical Solutions USA Inc., Chicago, IL USA; 4 Cardiovascular Center and Division of Cardiology, Department of Internal Medicine, National Taiwan University Hospital, Taipei, Taiwan; 5 Department of Surgery, National Taiwan University Hospital, Taipei, Taiwan; 6 Department of Medical Imaging and Radiological Technology, Yuanpei University of Medical Technology, Hsinchu, Taiwan

**Keywords:** Cardiovascular magnetic resonance, Extracellular volume fraction, Hematocrit, T1 mapping

## Abstract

**Background:**

Cardiovascular magnetic resonance (CMR)–derived extracellular volume (ECV) requires a hematocrit (Hct) to correct contrast volume distributions in blood. However, the timely assessment of Hct can be challenging and has limited the routine clinical application of ECV. The goal of the present study was to evaluate whether ECV measurements lead to significant error if a venous Hct was unavailable on the day of CMR.

**Methods:**

109 patients with CMR T1 mapping and two venous Hcts (Hct_0_: a Hct from the day of CMR, and Hct_1_: a Hct from a different day) were retrospectively identified. A synthetic Hct (Hct_syn_) derived from native blood T1 was also assessed. The study used two different ECV methods, (1) a conventional method in which ECV was estimated from native and postcontrast T1 maps using a region-based method, and (2) an inline method in which ECV was directly measured from inline ECV mapping. ECVs measured with Hct_0_, Hct_1_, and Hct_syn_ were compared for each method, and the reference ECV (ECV_0_) was defined using the Hct_0_. The error between synthetic (ECV_syn_) and ECV_0_was analyzed for the two ECV methods.

**Results:**

ECV measured using Hct_1_ and Hct_syn_ were significantly correlated with ECV_0_ for each method. No significant differences were observed between ECV_0_ and ECV measured with Hct_1_ (ECV_1_; 28.4 ± 6.6% vs. 28.3 ± 6.1%, p = 0.789) and between ECV_0_ and ECV calculated with Hct_syn_ (ECV_syn_; 28.4 ± 6.6% vs. 28.2 ± 6.2%, p = 0.45) using the conventional method. Similarly, ECV_0_ was not significantly different from ECV_1_ (28.5 ± 6.7% vs. 28.5 ± 6.2, p = 0.801) and ECV_syn_ (28.5 ± 6.7% vs. 28.4 ± 6.0, p = 0.974) using inline method. ECV_syn_ values revealed relatively large discrepancies in patients with lower Hcts compared with those with higher Hcts.

**Conclusions:**

Venous Hcts measured on a different day from that of the CMR examination can still be used to measure ECV. ECV_syn_ can provide an alternative method to quantify ECV without needing a blood sample, but significant ECV errors occur in patients with severe anemia.

## Introduction

The longitudinal relaxation time (T1) before and after the administration of contrast agents has been used to quantify the fraction of extracellular volume (ECV), which represents the extent of the extracellular space and can be used as a surrogate for quantifying diffuse myocardial fibrosis [1–4]. An ECV measurement requires a hematocrit (Hct) measurement to correct for contrast volume distributions in blood. The Society for Cardiovascular Magnetic Resonance (SCMR) Consensus Statement recommends that Hcts should be measured within 24 h of the CMR scan [5]. In many clinical practices, patients must have had a renal function evaluation within 3 months before cardiovascular magnetic resonance (CMR) with gadolinium contrast administration, from which a laboratory blood-derived (venous) Hct can be performed. In patients without laboratory bloodwork, an estimated glomerular filtration rate (eGFR) from blood drawn approximately 3 days before CMR can be alternatively used. To achieve accurate ECV measurements, a repeat venipuncture to acquire a Hct is performed on the day of CMR. Therefore, obtaining a venous Hct on the same day of the CMR is possible, but requires additional effort and increases the complexity of translating ECV quantification into routine clinical practice. As mentioned previously, Hct should be measured within 24 h of CMR as it may change over time. However, Hcts also vary with body posture [6] and diurnal fluctuation [7]. Studies have also demonstrated that Hct exhibits hour-to-hour, day-to-day, and even seasonal within-individual fluctuations [8, 9]. It remains unknown whether these variations lead to significant errors in ECV measurements.

Blood consists of two water-containing compartments, erythrocytes and plasma, and the fraction of water in these two compartments is roughly proportional to their fractional volume and is referred to as the Hct. The longitudinal relaxation rate (R1 = 1/T1) in blood has been shown to be linearly correlated with Hct [10–13]. Previous studies have proposed that the “synthetic” Hct (Hct_syn_) can be estimated from the native blood T1 values [14–16]. Treibel [17] and Fent [18] further demonstrated that synthetic ECVs (ECV_syn_) derived from synthetically calculated Hcts (Hct_syn_) demonstrated a strong correlation with ECVs derived from venous Hcts. However, several recent studies have suggested that using ECV_syn_ can result in the miscategorization of individual patients [19] and lead to clinical errors using 3T CMR [20]. Therefore, whether ECV_syn_ measurements using Hct_syn_ are feasible for clinical practice remains controversial and needs to be investigated further.

CMR-derived ECV can be evaluated using the region of interest (ROI)-based method either from native and postcontrast T1 maps or directly from ECV mapping. Whether the ECV value determined from the inline ECV mapping is comparable to the conventional T1 maps approach has yet to be established. Therefore, the goals of the present study were to evaluate: (1) whether significant ECV errors were found when the Hct was unavailable on the day of CMR, and (2) whether ECV values determined from the inline ECV method is comparable with that of the conventional ECV method.

## Methods

### Patient populations

This study was approved by the institutional review board. All the study participants provided written informed consent. Eligible patients underwent CMR and had two venous Hct performed: one measured on the same day of CMR (Hct_0_) and the other measured on a different day (Hct_1_). Subjects with Hct_0_ only were defined as the derivation group. The linear equation between blood R1(1/T1) and Hct_0_ was derived in the derivation group and used to estimate Hct_syn_ and calculate a synthetic ECV. Exclusion criteria were uncontrolled arrhythmia, impaired renal function (eGFR < 30 ml/min/1.73 m^2^), or contraindications to CMR (e.g., implanted devices). Clinical and demographic information, including underlying cardiac diagnoses, were collected.

### Imaging acquisition

CMR was performed at 1.5T (Magnetom Aera, Siemens Healthineers, Erlangen, Germany) using a 30-channel-body coil and 32-channel spine coil. Myocardial T1 mapping was performed using an electrocardiogram (ECG)-triggered modified Look-Locker inversion recovery (MOLLI) pulse sequence, both before and 10–15 min after a 0.15 mmol/kg intravenous administration of a gadolinium-based contrast agent (Dotarem, Guerbet LLC, Princeton, New Jersey, USA). The MOLLI protocol used a 5(3)3 sampling scheme for native T1 mapping and a 4(1)3(1)2 sampling scheme for postcontrast T1 mapping. Scan parameters were as follows: TE/TR 1.14/2.7 ms; flip angle 35°; bandwidth 977 Hz/Px; minimum TI 125–150 ms; TI increment 80 ms; slice thickness 8 mm; iPAT factor (GRAPPA) 2. Inline ECV mapping was automatically generated from the native and postcontrast T1 maps using an investigational prototype [22, 23]. Three inline ECV maps were reconstructed using venous Hcts (Hct_0_, Hct_1_) and Hct_syn_. Three evenly spaced short-axis slices were sequentially acquired from the left ventricular (LV) base to the apex.

### Imaging analysis

**Fig. 1:**
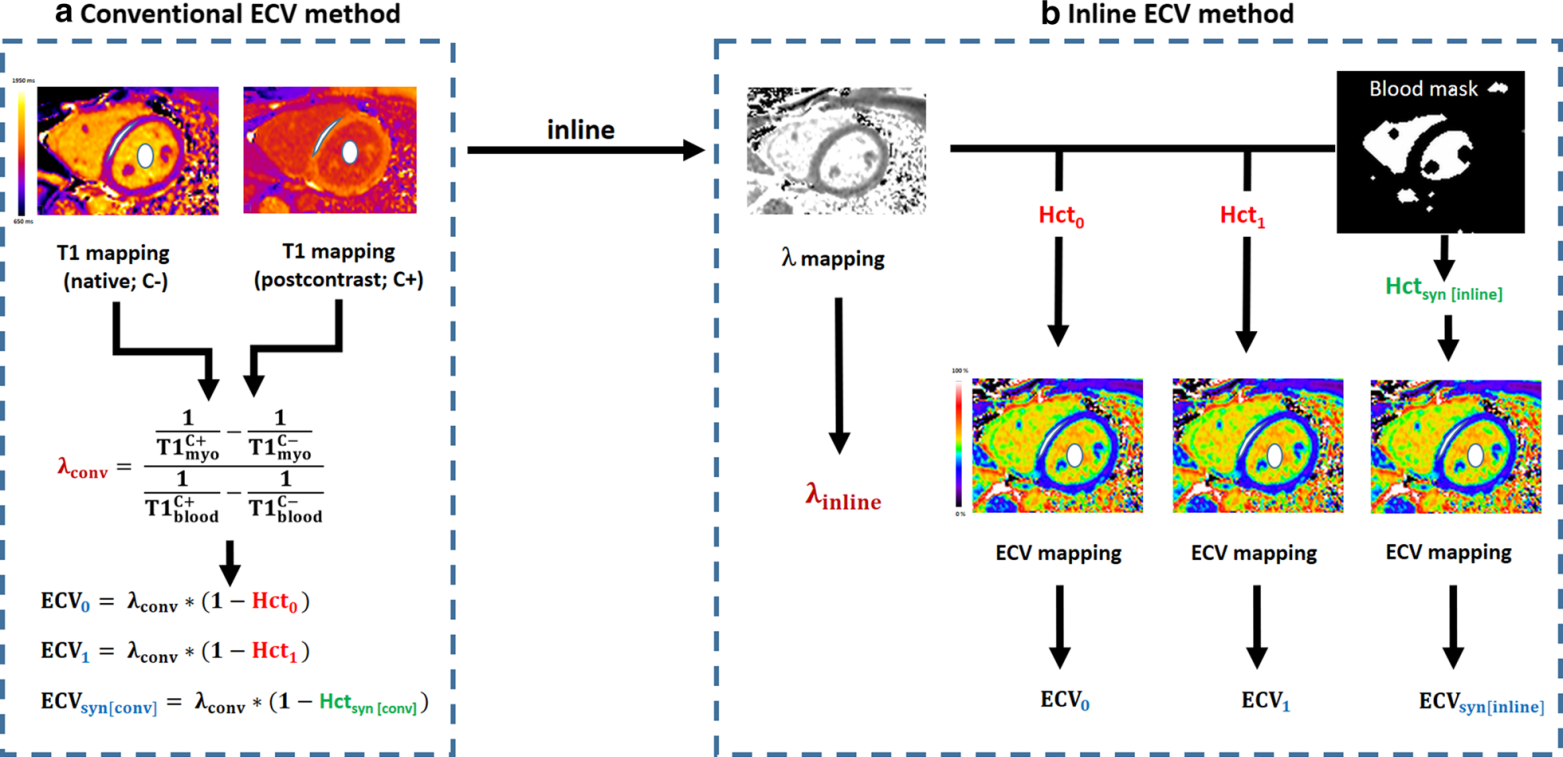
Schematic illustration of the conventional (**a**) and inline extracellular volume (ECV) methods (**b**). **a** The conventional ECV method derives a partition coefficient (λ_conv_) using averaged T1 values from regions-of-interests (ROIs) drawn in the myocardium and blood in native and postcontrast T1 maps. The ECV values are calculated from three different hematocrits (Hct). ECV_0_ is derived from an Hct_0_ measured on the same day as cardiovascular magnetic resonance (CMR) exam; ECV_1_ is derived using Hct_1_, which is measured on a different day; ECV_syn_ is derived using Hct_syn[conv]_ which is calculated from the native blood T1. **b** Inline partition coefficient (λ) mapping is automatically generated on a pixelwise basis after image registration of the native and postcontrast T1 maps. The blood mask is automatically generated from native T1 mapping and used to estimate Hct_syn[inline]_. Three inline ECV maps were reconstructed with Hct_0_, Hct_1_, and Hct_syn[inline]_. Three ECV values, ECV_0_, ECV_1_, and ECV_syn,_ are directly measured from three different inline ECV maps using the same myocardial ROI for T1 mapping

Commercial post-processing software (cvi42, Circle Cardiovascular Imaging, Calgary, Canada) was used to analyze ECVs. A flowchart of ECV quantification is illustrated in Fig. 1. ECV, measured from native and postcontrast T1 maps using a region-based method, was defined as the conventional ECV method. The ROIs in the blood and myocardium of the LV were drawn in the central area of the LV cavity and the septal myocardium on the T1-mapping image at the middle slice. The average T1 values of the segmented regions of interest were then computed. After obtaining the native and postcontrast T1 values, a partition coefficient (λ) was calculated by using the following formula [24]:

$$\lambda = \frac{{\frac{1}{{{\text{T}}1_{myocardium}^{postcontrast} }} - \frac{1}{{{\text{T}}1_{myocardium}^{native} }}}}{{\frac{1}{{{\text{T}}1_{blood}^{postcontrast} }} - \frac{1}{{{\text{T}}1_{blood}^{native} }}}}$$

The ECV values were then obtained by multiplying λ by 1-Hct. For inline ECV mapping, the native and postcontrast T1 maps were aligned using non-rigid motion correction and the above formula was applied for each pixel, resulting in a λ map. A 10% tolerance has been included for mismatching resolution/geometry, whereby the larger image was cropped to match the smaller image. The binary blood mask was automatically extracted using pre-defined thresholds and morphological imaging processing operations. The value for blood T1 was calculated as the median of all values of the T1 mapping identified by the blood mask. The native blood T1 was used to estimate Hct_syn_ with the local derivation model. The λ map were scaled by Hct_0_, Hct_1_, and Hct_syn_ to produce their respective ECV maps. Inline ECV values were measured directly from inline ECV maps using the same myocardial ROIs that were drawn on the T1 mapping in the conventional method (Fig. 1). The ECV measurement using Hct_0_ was defined as the reference ECV (ECV_0_) for both the conventional and inline ECV methods. ECV_0_ was compared with ECV derived from Hct_1_ (ECV_1_) and the ECV derived from Hct_syn_ (ECV_syn_) for each method. ECV_syn_ error was defined by differences in ECV using Hct_syn_ and Hct_0_. The partition coefficient estimated from the conventional ECV method (λ_conv_) was compared with those estimated from the inline ECV method (λ_inline_). The relative changes in Hcts were assessed by comparing Hct_0_ with Hct_1_ and Hct_syn_.

### Statistical analysis

Continuous variables were expressed as the means and standard deviations (SDs) or as median (IQR; interquartile range) as appropriate, and categorical variables were expressed as percentages. Correlations between the continuous variables were assessed with the use of the Pearson correlation coefficient. Bias and precision were evaluated with Bland–Altman analyses. Agreements between the measurements were assessed via the intraclass correlation coefficient (ICC) with a two-way random-effects model. Comparisons between values were made using a paired *t*-test for continuous values with normal distributions and a Wilcoxon signed-rank test for continuous values with non-normal distributions. Statistical tests were two-tailed, and a statistical significance was defined as p < 0.05. Type II error (β) was calculated and statistical power was estimated by 1-β for each comparison. Equivalence analysis was performed to assess whether ECV estimated by Hct_1_ and Hct_syn_ were similar with Hct_0_ for each method. Sample size calculation for equivalence analysis was evaluated to achieve a 5% two sided type I error and 90% statistical power [25]. Two-sided 95% confidential interval (CI) for the ECV difference between the two Hcts was used to compare with equivalence margin. Data were analyzed with SPSS (version 26, Statistical Package for the Social Sciences, International Business Machines, Inc., Armonk, New York, USA) and GraphPad Prism software (version 5.01, GraphPad Software, Inc., La Jolla, California, USA).

## Results

### Patient characteristics

**Fig. 2:**
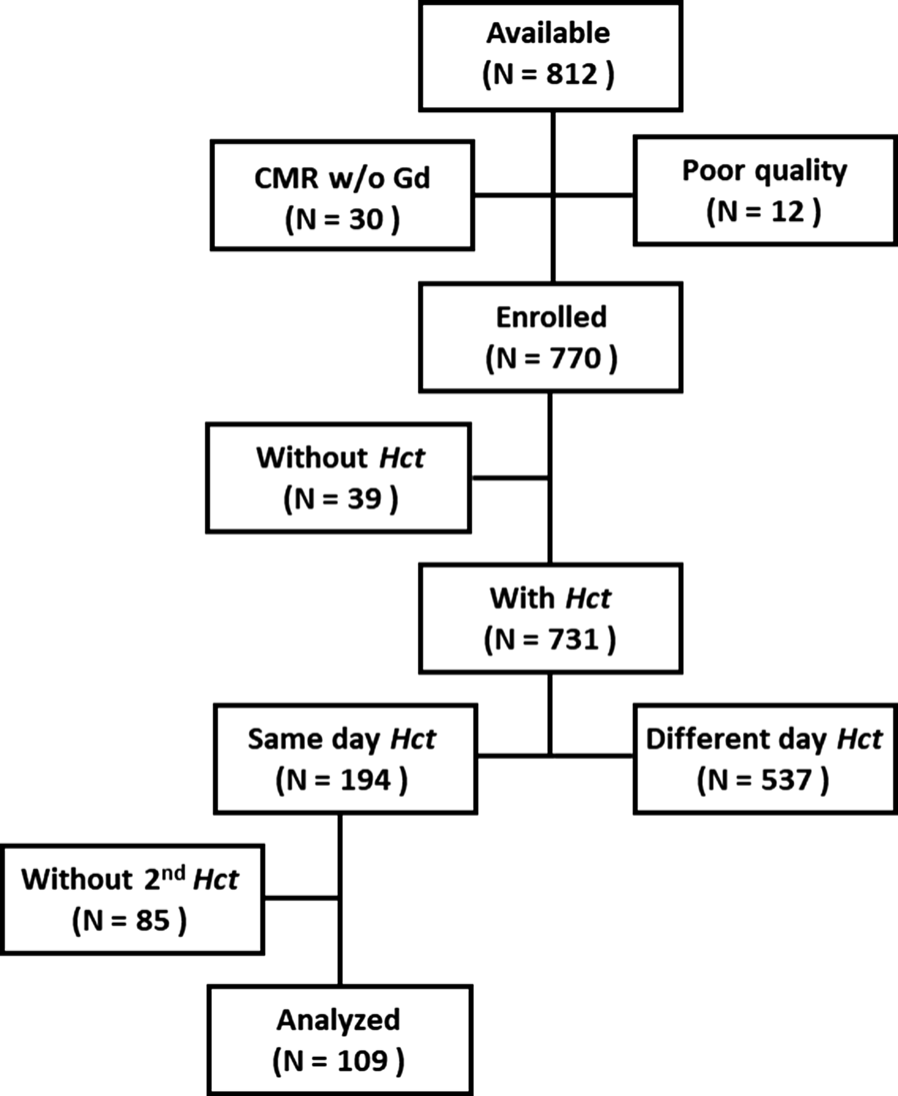
A flow chart showing the selection process in this study. 812 consecutive patients were available for inclusion in this cohort. A total of 770 subjects were included in this study; 37 patients that did not have hematocrit (Hct) data were excluded. 149 patients had both Hct data and CMR performed on the same day. Among these patients, 109 patients had a second Hct (HCT_1_) measure and were included for further analysis

812 consecutive patients were available for inclusion between March 2018 and May 2020. We excluded patients with suboptimal image quality (n = 12) and no contrast indication (n = 30) according to the exclusion criteria. A total of 770 subjects were included in this study. In this cohort, 37 patients without Hct data (5% in total) were excluded. One hundred and ninety-four patients had Hct data and CMR examinations performed on the same day (25.2% in total). Among these patients, 85 patients without a second Hct were used to derive local derivation model for Hct_syn_ and 109 patients who had a second Hct performed were included for further analysis (Fig. 2). The demographics of the study population are summarized in Table 1. The time interval between the date of the second Hct and CMR was 117 days (IQR: 27–274 days). All patients were outpatient referrals. 24 patients (22%) were hospitalized and 16 patients (17%) underwent interventional procedures between the date of the second Hct and CMR. However, there was no significant difference in blood pressure and heart rate between these two time points. Clinical diagnoses consisted of amyloidosis (n = 3, 2.8%), Brugada syndrome (n = 6, 5.5%), coronary artery disease (n = 12, 11%), heart failure (n = 6, 5.5%), dilated cardiomyopathy (n = 3 2.8%), Anderson-Fabry disease (n = 4, 3.7%), hypertrophic cardiomyopathy (n = 12, 11%), myocarditis (n = 1, 0.9%), hypertensive cardiomyopathy (n = 29, 27%), tetralogy of Fallot (n = 6, 5.5%), and suspected cardiovascular disease (n = 18, 17%).

**Table 1:** Patient characteristics

	N or mean ± SD	% or range
Age (years)	53 ± 19	17–89
Gender		
Male	66	62
Female	43	38
BSA (m^2^)	1.73 ± 0.25	1.37–2.24
Heart rate (bmp)	74 ± 15	43–153
LV function		
EDVI (ml/m^2^)	60.5 ± 20.3	32.0–156.9
ESVI (ml/m^2^)	17.6 ± 15.1	3.3–101.9
EF (%)	73 ± 13	33–93
Diagnosis		
Amyloidosis	3	2.8
Brugada Syndrome	6	5.5
Coronary artery disease	12	11
Heart failure	6	5.5
Dilated cardiomyopathy	3	2.8
Anderson-Fabry disease	4	3.7
Hypertrophic cardiomyopathy	12	11
Hypertensive cardiomyopathy	29	27
Myocarditis	1	0.9
Tetrology of Fallot	24	5.5
Others CVD	18	17

*BSA* body surface area, *EDVI* end-diastolic volume index, *ESVI* end-systolic volume index, *EF* ejection fraction, *Hct* hematocrit, *LV* left ventricular, *CVD* cardiovascular disease

### Local derivation model for synthetic hematocrit

**Fig. 3:**
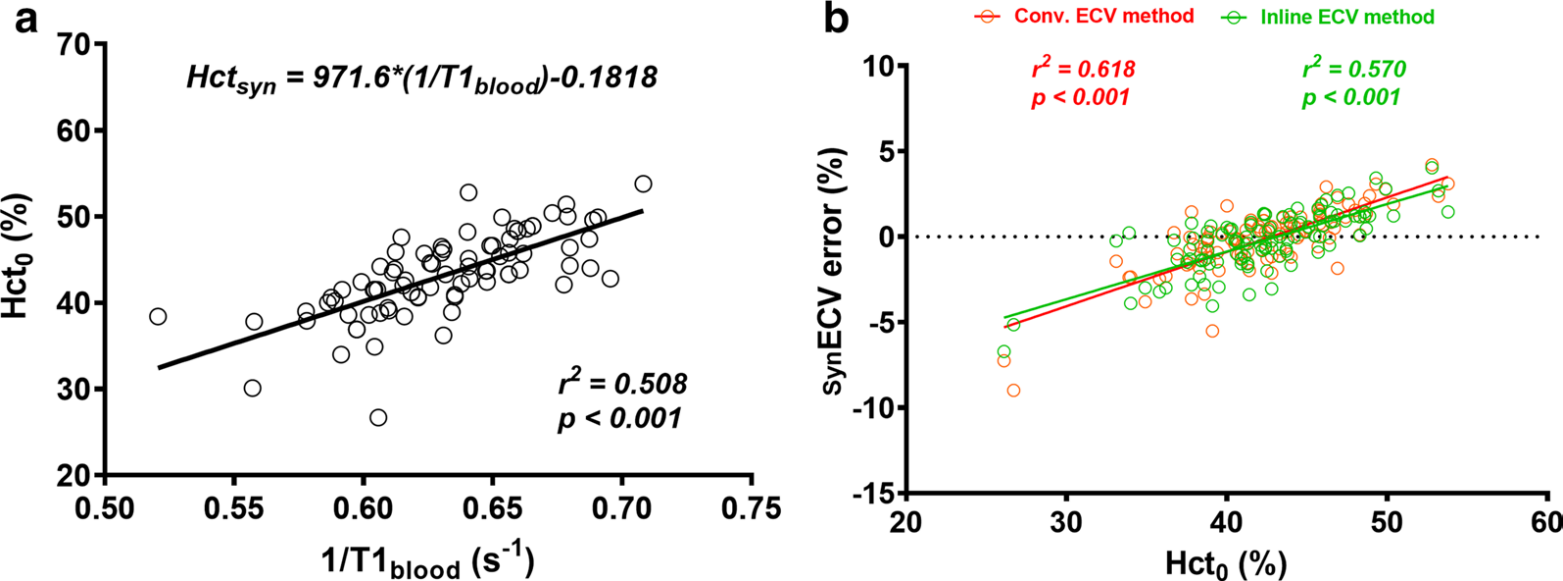
**a** Linear regression between native blood 1/T1 (R1) and Hct measured on the same day as that of CMR (Hct_0_). **b** Correlation graph comparing synthetic ECV error (synECV_error_) obtained from the conventional ECV method (red line) and the inline ECV method (black line). Both errors are positively associated with the Hct levels

The regression between Hct_0_ and native T1_blood_ was linear (R^2^ = 0.51, p < 0.001), and the regression equation was Hct_syn_ = [971.6*(1/T1_blood_)] + 0.1818 (Fig. 3a) in the derivation group.

### ECV comparisons with differently measured hematocrits and quantification methods

**Fig. 4:**
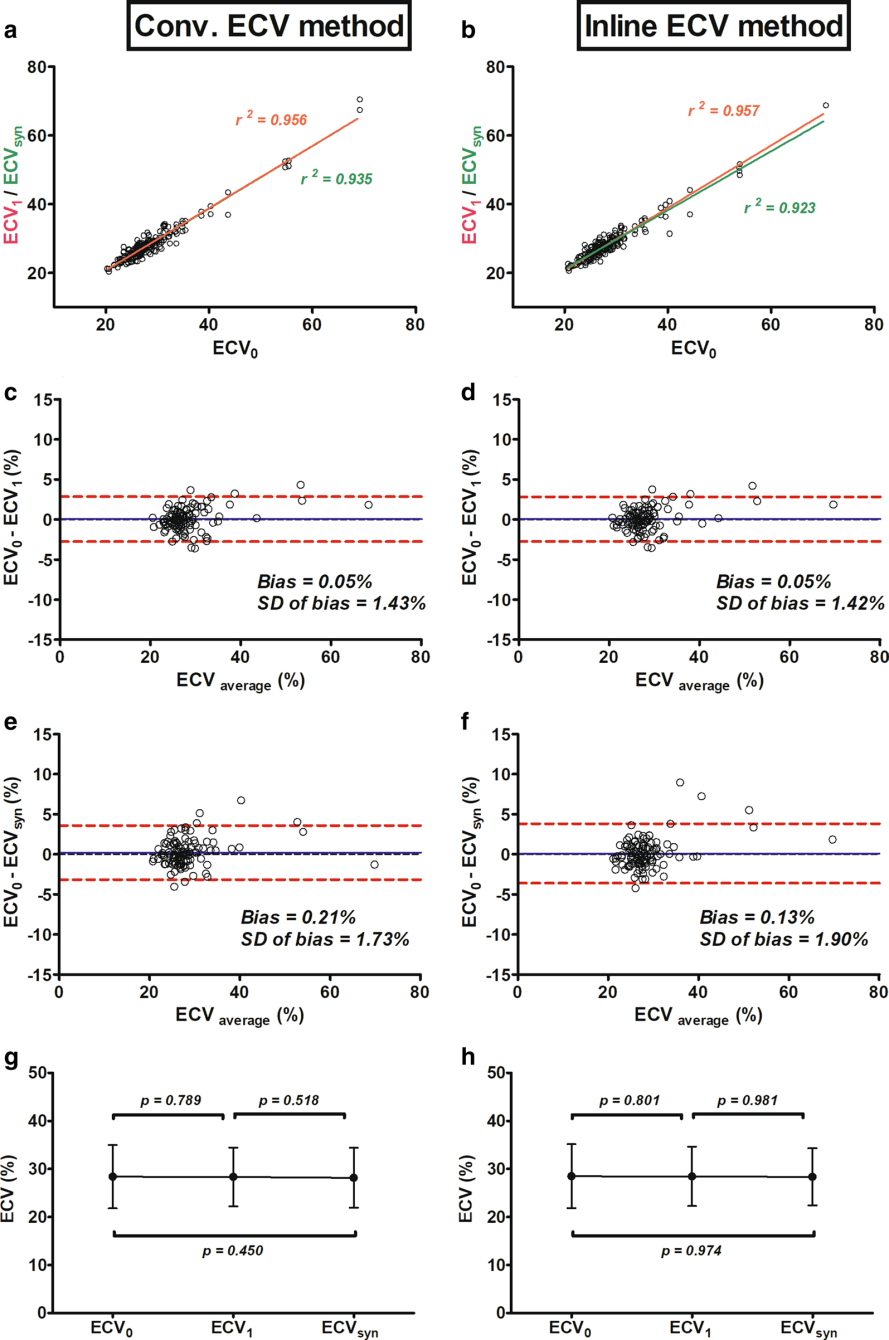
Correlation and comparisons among three extracellular volumes (ECVs) derived from three different hematocrits (Hcts) using the conventional ECV and the inline ECV methods. ECV_1_ and ECV_syn_ are both strongly correlated with ECV_0_ in the conventional method (**a**) and the inline ECV method (**b**). In the conventional method, Bland–Altman plots indicate minimal bias between ECV_1_ and ECV_0_ (**c**) and between ECV_syn_ and ECV_0_ (**e**). Similarly, Bland–Altman plots also indicate minimal bias between ECV_1_ and ECV_0_ (**d**) and between ECV_syn_ and ECV_0_ (**f**) in inline ECV method. No significant differences among 3 ECVs in the conventional method (**g**) and the inline ECV method (**h**) were observed. ECV_0_ is derived using an Hct obtained on the day of CMR (CMR; Hct_0_), ECV_1_ is derived with Hct_1_ is measured from a different day than CMR, and ECV_syn_ is derived with Hct_syn_, which is calculated from native blood T1 mapping

Using the conventional method, ECVs measured with Hct_1_ and Hct_syn_ were significantly correlated with ECV_0_ (Fig. 4a). The coefficient of determination (*r*^2^) between ECV_0_ and ECV_1_ was 0.956 (p < 0.001); the *r*^2^ between ECV_0_ and ECV_syn_ was 0.935 (p < 0.001). A Bland–Altman analysis indicated a 0.05% bias (− 2.8 to 2.9%, Fig. 4c) between the ECV_0_ and ECV_1_ and a 0.2% bias (− 3.2 to 3.6%, Fig. 4e) between the ECV_0_ and ECV_syn_. The ICC coefficient between ECV_0_ and ECV_1_ was 0.987 with a 95% CI 0.991–0.982, and between ECV_0_ and ECV_syn_ was 0.981with a 95% CI 0.987–0.973. These results showed no significant differences between ECV_0_ and ECV_1_ (28.4 ± 6.6% vs. 28.3 ± 6.1%, p = 0.789, β = 0.211) and ECV_0_ and ECV_syn_ (28.4 ± 6.6% vs. 28.2 ± 6.2%, p = 0.450, β = 0.536; Fig. 4g).

Using the inline method, ECVs measured with Hct_1_ and Hct_syn_ were also significantly correlated with ECV_0_ (Fig. 4b). The coefficient of determination (*r*^2^) between ECV_0_ and ECV_1_ measured with the inline ECV method was 0.957 (p < 0.001); the *r*^2^ between ECV_0_ and ECV_syn_ was 0.923 (p < 0.001). Bland–Altman analysis indicated a 0.05% bias (− 2.73 to 2.82%, Fig. 4d) between ECV_0_ and ECV_1_, and a 0.1% bias (− 3.58 to 3.85%, Fig. 4f) between ECV_0_ and ECV_syn_. The ICC coefficient between ECV_0_ and ECV_1_ was 0.988 with a 95% CI 0.992–0.982, and between ECV_0_ and ECV_syn_ values was 0.977 with a 95% CI 0.984–0.966. These results showed that in using the inline ECV method there was no significant difference between ECV_0_ and ECV_1_ (28.5 ± 6.7% vs. 28.5 ± 6.2, p = 0.801, β = 0.199) and ECV_0_ and ECV_syn_ (28.5 ± 6.7% vs. 28.4 ± 6.0, p = 0.974, β = 0.026) (Fig. 4h).

In addition, the partition coefficient measured from the conventional ECV method was also not different with that measured from the inline ECV method (49.4 ± 11.4% vs. 49.5 ± 11.4%, p = 0.620, β = 0.378).

### Hematocrits compared on different sampling days and derived from native blood T1 mapping

**Fig. 5:**
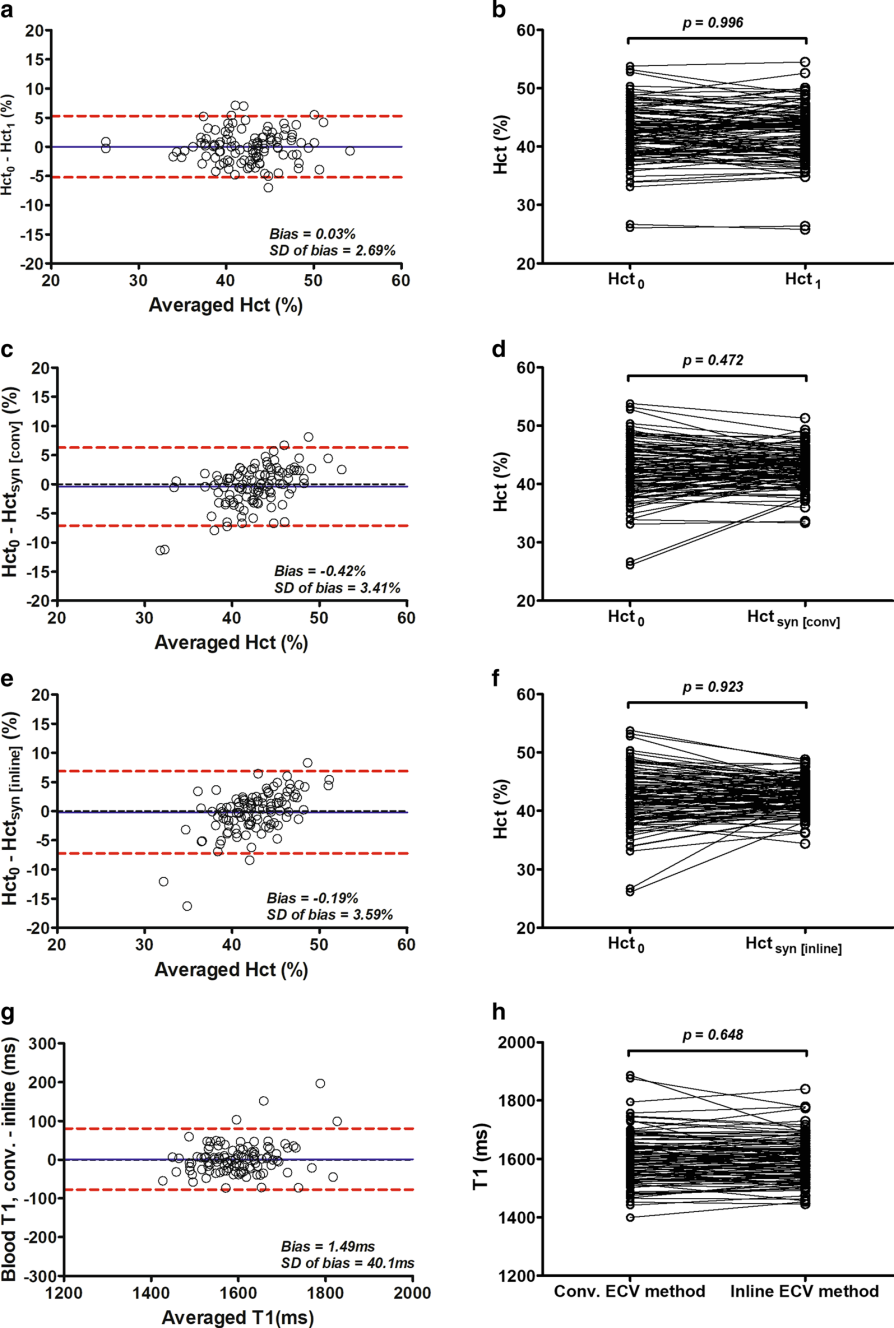
Comparisons among three different hematocrits (Hcts) and between two native blood T1. Bland–Altman plots indicate the minimal bias between Hct_1_ and Hct_0_ (**a**), between Hct_syn [conv]_ and Hct_0_ (**c**), and between Hct_syn [inline]_ and Hct_0_ (**e**). There is no statistical difference between Hct_1_ and Hct_0_ (**b**), between Hct_syn [conv]_ and Hct_0_ (**d**), and between Hct_syn [inline]_ and Hct_0_ (**f**). Bland–Altman plot indicates a 1.5 ms bias and confidence limit (7.2–6.9 ms) using the inline method compared with using the conventional method (**g**), which resulted in no statistical difference between the conventional and inline ECV methods for native blood T1 measurements (**h**). Hct_0_ is Hct obtained on the same day as theCMR. Hct_1_ is Hct obtained on a different day from the CMR. Hct_syn [conv]_ and Hct_syn [inline]_ are Hcts obtained synthetically from native blood T1mapping using the conventional and inline ECV methods, respectively

Bland–Altman analysis indicated a 0.03% bias (− 5.2 to 5.3%) between Hct_1_ and Hct_0_ (Fig. 5a), resulting in no significant difference between these two variables (42.3 ± 4.7% vs. 42.4 ± 4.7%, p = 0.996, β = 0.004; Fig. 5b). Regarding Hct_syn_ derived from the two different methods, Bland–Altman analysis indicated a − 0.4% bias (− 7.1 to 6.3%) using the conventional ECV method (Fig. 5c) and a − 0.2% bias (− 7.2 to 6.9%) using the inline ECV method (Fig. 5e). These results showed that Hct_syn_ was not statistically different than Hct_0_ using the conventional ECV method (42.8 ± 3.1% vs. 42.4 ± 4.7%, p = 0.472, β = 0.410) (Fig. 5d) and using the inline ECV method (42.5 ± 2.8% vs. 42.4 ± 4.7%, p = 0.923, β = 0.072; Fig. 5f). Since the Hct_syn_ was determined from native blood T1 measurements, we further compared blood T1 measured using these two methods. From Bland–Altman analysis, there was a 1.5 ms bias and confidence limit (− 7.2 to 6.9 ms) using the inline method compared with using the conventional method (Fig. 5g), which resulted in no statistical difference between the conventional and inline ECV methods for native blood T1 measurements (1599 ± 84 ms vs. 1598 ± 75 ms, p = 0.648, β = 0.351; Fig. 5h). Hct comparsions were listed in Table 2.

**Table 2:** Comparison of venous and synthetic hematocrits

	Mean ± SD	Range
Hct_0_ (%)	42.4 ± 4.7	(26.1, 53.8)
Hct_1_ (%)	42.3 ± 4.7	(25.8, 54.5)
Hct_syn [conv]_ (%)	42.8 ± 3.1	(33.4, 51.3)
Hct_syn [inline]_ (%)	42.5 ± 2.8	(34.4, 48.8)

Hct_0_ is hematocrit (Hct) obtained on the same day as that of CMR. Hct_1_ is Hct obtained on a different day from that of CMR. Hct_syn [conv]_ and Hct_syn [inline]_ are Hcts obtained synthetically from native blood T1mapping using the conventional and inline ECV methods, respectively

### Equivalence analysis for ECV differences

**Fig. 6:**
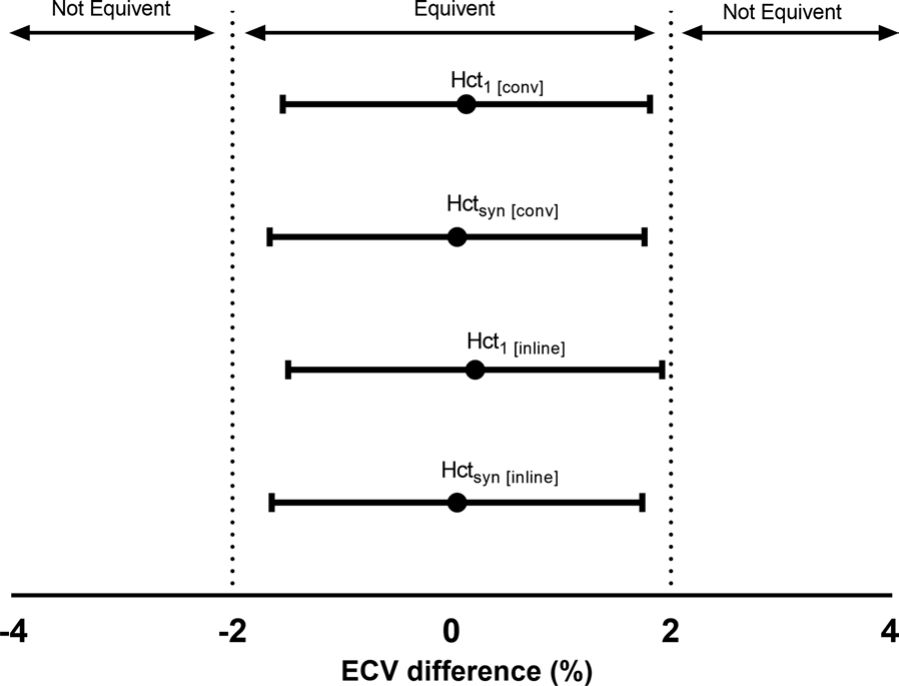
Interpretation of equivalence of tested hematocrits (Hct_1_, Hct_syn_) to the standard hematocrit (Hct_0_) for ECV measurements. The dashed vertical lines indicate the predefined equivalence margins (− 2 to 2%) and the horizontal lines represent the 95% confidence interval (CI) of the difference in ECV between compared Hcts. Equivalence is concluded if the entire CI is within the equivalence margins. Hct_1 [conv]_ and Hct_1 [inline]_ are hematocrits obtained on a different day from that of CMR f using the conventional and inline ECV methods, respectively. Hct_syn [conv]_ and Hct_syn [inline]_ are Hcts obtained synthetically from native blood T1 mapping using the conventional and inline ECV methods, respectively

Normal myocardial ECV in normal subjects has been reported at 1.5T as 25.3 ± 3.5% (n = 81) [26] and 25.4 ± 2.5% (n = 62) [23], respectively. Therefore, a ± 2% defined equivalence margin was considered acceptable in this study. Assuming SD of the ECV difference between the two Hcts was 4%, 207 total samples were needed to achieve a 0.90 power with α = 0.05 for the equivalence study. The two-sided 95% CI was calculated as followed:

$m1-m2 \pm 1.96\sqrt{\frac{{\sigma }_{1}^{2}+{\sigma }_{2}^{2}}{n}},$where m1 and m2 were means; σ_1_ and σ_2_ were SDs in the tested ECV and ECV_0_, respectively, and the n was the sample size (n = 109). Figure 6 showed the entire CI of ECV differences were within the equivalent margin in both Hct_1_ and Hct_syn_ for each method.

### Synthetic extracellular volume error is associated with hematocrit levels

The error associated with ECV_syn_ measurements obtained from the two methods is shown in Fig. 6. Both errors were positively associated with Hct_0_ levels, which suggest that ECV_syn_ could be underestimated at lower Hcts and overestimated at higher Hcts. Based on a sub-analysis of current data, the cut-off value for Hct_syn_ was determined on the error of ECV_syn_ was less than 2%. The cut-off range for Hct_syn_ was obtained from 36.5 to 49.0% using the conventional method, and from 36.0 to 50.4% using the inline method, respectively.

## Discussion

In this study, we compared ECV measurements with three different Hcts and two ECV methods. ECV measured with venous Hct drawn on a different day from that of CMR (ECV_1_) was significantly correlated with reference ECV (ECV_0_) using both conventional and inline ECV methods. Minimal biases and limited discrepancies were also found. These findings demonstrate that for both methods, venous Hcts could be used to estimate ECV, and that it did not matter if the Hct was from the day of CMR or from a different day. In addition, a significant correlation and good agreement between ECV_syn_ and ECV_0_ were noted in both methods. These results suggest that ECVs estimated with native blood T1 could be used for an ECV measurement if venous Hct was unavailable. Moreover, the equivalence analysis indicated that 95% CI of ECV differences between the two Hcts lay entirely within the equivalent margin for both methods. These results further strengthen our findings. The partition coefficient determined from the inline ECV method showed no significant difference with that of the conventional ECV method, suggesting that ECV measured using the inline method is reliably compared with ECV measured using the conventional method. Hct are necessary for ECV calculations and have been reportedly shown to be influenced by body posture [6] and diurnal fluctuation [7]. Some studies have also demonstrated that hemoglobin exhibits hour-to-hour, day-to-day, or even seasonal within-individual fluctuations [8, 9]. Thirup et al. [27] performed a meta-analysis to explore the substantial variation of Hcts looking at 12 studies representing 638 healthy adults that had sampling intervals of 1 day to 1–2 months. They reported that both normal within-subject variation and analytical variation were 3%, resulting in an approximately 12% relative change between the two successive Hct measurements. Theoretically, a 1% change in Hct would lead to a 0.67% change in the ECV. Based on this reference, a 12% change in Hct could cause an 8% difference in the ECV. In this study, we demonstrated that there was no significant difference in the measured Hcts and ECVs using Hcts obtained on the same day vs. on a different day from the CMR, which could imply that the day on which an Hct is obtained does not have a significant effect on ECV measurements.

Hct_syn_ can be derived from the native blood T1 values and used to calculate ECV_syn_ without needing blood sampling. Treibel et al. reported that ECV_syn_ measured from Hct_syn_ provide a validated, noninvasive quantification of the myocardial extracellular space without the need for a blood sample [17]. Fent et al. further demonstrated that ECV_syn_ values strongly correlated with conventionally measured ECV at both 1.5T and 3T [18]. In contrast, Raucci et al. proposed that using ECV_syn_ might result in a clinically significant miscategorization of pediatric and young adult patients [19]. In addition, Shang et al. further demonstrated that ECV_syn_ could lead to clinical errors using 3T CMR, suggesting that the use of ECV_syn_ could incorrectly categorize 6–25% of patients [20]. Consistent with some studies, our results showed that the ECV_syn_ was strongly correlated with the ECV_0_ and revealed no significant difference with ECV_0_ using both methods.

Our results showed that the native blood T1 values measured using the inline ECV method were not significantly different from those measured using the conventional ECV method. This finding suggests that automated blood region segmentation is reliable to estimate the blood T1 compared to conventional ROI method. Despite the blood T1 calculation, several factors that could influence native blood T1 are possible, such as physiologic variation (e.g., hemoglobin oxygenation [28], serum total proteins [29] and temperature), and technical issues (e.g., the efficiency of the inversion pulse, the pulse sequence parameters, the magnetization transfer effects, the magnetic field heterogeneity, and the fitting algorithms) [30–33]. In this study, we demonstrated that for the two ECV methods, ECV_syn_ error was significantly correlated with Hct, and the relatively large ECV_syn_ errors occurred when Hcts were lower compared with when they were higher. These findings suggest that the derived coefficients for Hct_syn_ were only confidently applied to the range in which it was derived and extrapolations that occurred outside of the range resulted in less confident estimates. The patients with very low Hct values could have other blood composition abnormalities that also affect T1 measurements. For these patients, Hct_syn_ and ECV_syn_ should be interpreted with caution, and a venous Hct should be used, if possible. For patients without a timely assessment of Hct, our results demonstrated that the last available Hct is feasible for use in ECV measurements. We defined the cut-off range (36–50%) for Hct_syn_ based on the ECV_syn_ error of < 2%. This range is similar with normal range of Hct: 41–50% for men and 36–48% for women. Although ECV_syn_ could lead to ECV error in patients with severe anemia, it also provides an alternative method to assess ECVs if venous Hcts are unavailable.

The purposes of CMR exams were diverse in this retrospective cohort. Patients with Hct_0_ were primarily intended for ECV evaluation. Therefore, only 25.2% of the enrolled CMR patients had Hct data from the same day as CMR, and 5% of patients had no Hct data before CMR. For our institution, two steps were required to obtain Hct_0_ in our workflow. First, the laboratory bloodwork was requested with “fractional blood sampling”, with one fraction for estimated eGFR and the other for Hct, so that two laboratory exams were tested simultaneously. Second, patients went to the laboratory department in advance to register for Hct evaluation and brought a blood collection tube to the CMR exam room. Then the blood sample was taken during the peripheral intravenous insertion by a CMR nurse before the exam. Due to number of additional steps required, this resulted in an 80% success rate for Hct_0_ collection in our experience. Common points of failure were the physician’s assistant forgetting to remark “fractional blood sampling” on laboratory order sheet, the laboratory technologist omitting the request for two laboratory exams and blood sample not being taken during peripheral intravenous insertion. As Hct_0_ collection is not part of our routine workflow, good communications between different departments and keeping patients well-informed were important for ensuring that Hct can be obtained on the same day as the CMR exam.

The conventional ECV method is most often used to quantify diffuse myocardial fibrosis. In these cases, the ROI can be drawn within the myocardium, avoiding the enhanced regions shown on late gadolinium enhancement (LGE) imaging. This method assumes that diffuse fibrosis is distributed homogeneously (uniform ECV) within the non-infarcted regions. However, the spatial variation of diffuse fibrosis is diverse and depends on various cardiomyopathies [34]. Therefore, ECV measurements could be affected by the position of an ROI if any significant regional variation exists in the fibrotic areas. Compared with conventional ECV method, inline ECV mapping not only allowed for ECV measurements at the time of CMR examination, but it also provided an ability to assess the heterogeneity of the myocardial tissue. This approach is clinically desirable and could potentially be used to identify subtle differences in myocardial ECVs earlier. In this study we demonstrated that the partition coefficient determined from the inline ECV method was not significantly different with that measured from the conventional ECV method. This result suggests that inline ECV method offers an identical ECV quantification compared to the conventional ECV method.

## Study limitations

There are several limitations to our study. First, the ECVs measured using an Hct from a different day from that of CMR were not significantly different from those measured on the same day. This result could be associated with the lack of a statistical difference in the Hct depending on the time between when the measured Hct and CMR occurred. Although Hct has substantial variation, this variation does not appear to influence ECV measurements. Second, our study was performed with a single T1 pulse sequence (MOLLI), however different sequences of T1 mapping have been reported to yield different absolute ECV values [35]. Third, imaging quality for the ECV mapping was not evaluated in this study. Whether this comparison is identical with different pulse sequences or depends on the quality of ECV mapping is unknown and needs further investigation. Finally, all data were acquired at a single institution using a single CMR vendor. Multi-center studies, including larger numbers of patients, with different vendors should be further performed to validate these results.

## Conclusion

Our study demonstrated that venous Hct measured on a different day than that of CMR is still useful for the calculation of ECVs regardless of the quantification method. ECV_syn_ values could provide an alternative method to quantify ECVs without requiring a blood sample but significant error may occur when patients have either extremely low or high Hcts. Inline ECV mapping could provide a method to quickly detect myocardial tissue heterogeneity and measure ECV abnormalities without the timely presence of a Hct.

## Data Availability

The datasets used and/or analyzed during the current study are available from the corresponding author upon reasonable request.

## Abbreviations

CMR: Cardiovascular magnetic resonance
ECG: Electrocardiography
ECV: Extracellular volume fraction
ECV_0_: Reference ECV
ECV_syn_: Synthetic ECV
eGFR: Estimated glomerular filtration rate
Hct: Hematocrit
Hct_0_: Reference hematocrit
Hct_syn_: Synthetic hematocrit
ICC: Intraclass correlation
LGE: Late gadolinium enhancement
LV: Left ventricle/left ventricular
MOLLI: Modified Look-Locker inversion recovery
ROI: Region of interest
SCMR: Society for Cardiovascular Magnetic Resonance
SD: Standard deviation
T1: Longitudinal relaxation time
